# Physical Performance of Geriatric Women and Its Impact on Fracture Risk and Bone Mineral Density Assessed with Radiofrequency Echographic Multispectrometry (REMS)

**DOI:** 10.3390/life14121579

**Published:** 2024-12-01

**Authors:** Elena Bischoff, Stanislava Popova-Belova, Fabian Bischoff, Nikola Kirilov

**Affiliations:** 1 Department of Health Care, Faculty of Medicine, Trakia University, 6007 Stara Zagora, Bulgaria; 2 Medical University of Plovdiv, Medical Faculty, Department of Propedeutic of Internal Diseases, General Hospital “Sveti Georgi”, 4002 Plovdiv, Bulgaria; 3 IPSMP Rheumatology, 6000 Stara Zagora, Bulgaria; 4 Institute of Medical Informatics, Heidelberg University Hospital, 69120 Heidelberg, Germany

**Keywords:** physical performance, geriatric women, fracture risk, bone mineral density, REMS

## Abstract

Background: This study aimed to investigate the impact of physical performance of geriatric women on their fracture risk and bone mineral density (BMD) assessed with radiofrequency echographic multispectrometry (REMS). Methods: We conducted a prospective observational study to assess the physical performance, BMD and fracture risk in 182 geriatric women aged 60 years and older. BMD was measured using REMS scanning (developed by Echolight S. p. a., Lecce, Italy), and the Fracture Risk Assessment Tool (FRAX) was utilized to estimate fracture risk. Physical performance was assessed using hand grip strength (HGS), Timed Up and Go (TUG), Five Times Sit-to-Stand Test (5XSST) and Flamingo test. Results: The mean HGS of both hands differed significantly between the groups with normal BMD, osteopenia and osteoporosis measured at the lumbar spine and hip. The mean TUG time of the subjects with osteoporosis was significantly higher (13.77 s) than those with osteopenia (7.14 s) and normal BMD (6.05 s) of the hip (*p* = 0.024). The mean 5XSST time of the subjects with normal BMD (8.86 s) was lower than those with osteopenia (9.30 s) and osteoporosis (13.6 s) of the hip (*p* = 0.012). Conclusions: This study revealed strong associations between physical performance and fracture risk. Rehabilitation programs focused on strength and mobility may be essential for fracture prevention.

## 1. Introduction

As the global population ages, osteoporosis and fractures pose a significant public health issue, especially among older women. Osteoporosis, marked by reduced bone mineral density (BMD) and tissue deterioration, raises fracture risk, leading to severe health complications. The World Health Organization (WHO) projects that by 2050, hip fractures will exceed 6 million annually, predominantly affecting women, due to menopause and hormonal changes [1,2,3].

Older women face unique challenges, with higher rates of osteoporosis and fractures compared to men, linked to lower peak bone mass, longer life expectancy and estrogen deficiency. Hip and spine fractures result in significant morbidity, mortality and decreased quality of life. A total of 20% of hip fracture patients require long-term care and lead to a high mortality rate in the first year [4,5,6,7].

Furthermore, the economic burden of osteoporosis-related fractures is substantial, impacting healthcare systems worldwide. Public health initiatives must prioritize education and awareness of osteoporosis and its consequences, emphasizing the importance of maintaining BMD through nutrition, physical activity and regular screenings [8].

Recent research underscores that skeletal health involves not just BMD but also bone quality, microarchitecture and various risk factors [9,10,11,12,13]. Additionally, other determinants of fracture risk include muscle strength, functional mobility and balance, all of which are crucial for maintaining physical stability and preventing falls. For instance, studies have shown that individuals with reduced muscle strength or impaired balance are at a heightened risk of falls, which can lead to fractures, even if their BMD measurements fall within the normal range [14,15,16]. Furthermore, lifestyle factors such as calcium and vitamin D intake as well as hormonal changes play an essential role [17,18,19]. Regarding fracture risk, there is a growing consensus in the medical community about the importance of comprehensive fracture risk assessments that incorporate not only BMD but also other clinical risk factors and assessments of functional status. Tools such as the Fracture Risk Assessment Tool (FRAX) integrate multiple variables to provide a more accurate estimate of fracture risk, thus enabling better-targeted prevention strategies [20].

Recent advancements in imaging technologies, such as radiofrequency echographic multispectrometry (REMS), which showed a strong agreement with dual-energy X-ray absorptiometry (DXA), provide a novel approach to evaluating bone quality and BMD [21,22]. REMS represents a significant evolution in the assessment of skeletal health, as it is a radiation-free, portable technology for the evaluation not only of BMD but also bone quality through a fragility score at central regions such as the lumbar spine and femoral neck [23]. Moreover, the portability and ease of use of REMS as well as accuracy in precision make it a useful tool in the management of osteoporosis as an alternative to DXA [24,25]. It can be utilized in various settings, from outpatient clinics to nursing homes, facilitating more widespread screening for osteoporosis and enabling timely interventions. This accessibility is particularly beneficial for geriatric populations, who may have difficulty accessing traditional imaging facilities [26,27].

The objective of this study was to assess the relationships between physical performance including by the hand grip strength (HGS), Timed Up and Go (TUG) test, Five Times Sit-to-Stand Test (5XSST) and Flamingo test with T-scores and fracture risks in a cohort of 182 geriatric women. The primary aim was to evaluate the associations between physical performance, T-score and fracture risk as assessed by FRAX. Specifically, this study sought to examine how age and BMI differed according to physical performance, whether the HGS, TUG and 5XSST results differed between the groups according to the diagnosis of normal BMD, osteopenia and osteoporosis, and whether physical performance correlated with the T-score and 10-year risk of major osteoporotic fractures (MOFs) and hip fractures (HFs). This research could provide insights into the role of physical performance in predicting bone health and fracture risk in geriatric women.

## 2. Materials and Methods

### 2.1. Study Design and Population

We conducted a prospective observational study to assess the physical performance, BMD and fracture risk in a cohort of 182 geriatric women aged 60 years and older. Participants were recruited from June 2023 to September 2024, signing an informed consent form prior to participation. Subjects were assessed at two centers—Trakia University and a rheumatology practice in Stara Zagora, Bulgaria. All assessments for each participant were completed on the same day. The study protocol was approved by the ethics committee of Trakia University, Stara Zagora, Bulgaria. Participants were divided into three age decades: 60–70 years, 71–80 years and over 80 years. The inclusion criteria were age of over 60 years, willingness to participate and sign the informed consent form, appropriate physical condition and no contraindications to carry out the physical performance tests, willingness to undergo the REMS scan either of the lumbar spine or hip, and according to the results, willingness to be classified as normal, osteopenic or osteoporotic, as well as willingness to answer the FRAX questionnaire. The exclusion criteria were the inability to perform the tests and medical conditions such as severe heart failure, coronary syndrome, arrhythmia, etc.

Demographic data, including age, weight, height, body mass index (BMI) and FRAX risk factors were collected. Weight was measured in kilograms (kg) using a calibrated scale and height was recorded in centimeters (cm) using a stadiometer. BMI was calculated using the following formula: BMI = kg/m^2^.

### 2.2. BMD and Fracture Risk

The portable ultrasound bone densitometer Echos Plus (device EchoS SN 001-230301), developed by Echolight S. p. a., a company in Lecce, Italy, was used to perform the scans. The BMD and T-score were measured through REMS technology acquired from the scans of the lumbar spine in 179 participants and of the femoral neck in 173 participants. In patients where it was not possible to measure more than one vertebra or where implants were present, only one anatomical site was assessed. The total REMS-based BMD of the lumbar spine was recorded in g/cm^2^. T-scores for both the lumbar spine and femoral neck were calculated from the REMS-based BMD values compared to a reference population and classified according to the following WHO criteria: normal— T-score > −1.0 standard deviation (SD), osteopenic: T-score between −1.0 and −2.5 SD, osteoporotic: T-score ≤ −2.5 SD.

FRAX was utilized to estimate the 10-year probability of major osteoporotic fractures (MOFs) and hip fractures (HFs). The mean FRAX values were computed along with their SDs and ranges.

### 2.3. Physical Performance

Physical performance was assessed using the hand grip strength (HGS), Timed Up and Go (TUG) test, Five Times Sit-to-Stand Test (5XSST) and Flamingo test.

HGS was evaluated using a Jamar hand dynamometer (model 081028950, company Patterson Medical, Reims, France). Isometric muscle contraction was measured by the device in kg for both the right and left hands in neutral position and 90° of elbow flexion three times and the mean value were recorded.

For the TUG test, participants were instructed to stand up from a chair, walk a distance of three meters, turn around, walk back to the chair and sit down. The time taken to complete this task was recorded in seconds (s). According to the TUG test, four groups were formed as the following: TUG < 10 s, TUG = 11–20 s, TUG = 21–29 s and TUG ≥ 30 s.

For the 5XSST, participants were asked to rise from a seated position and sit down five times as quickly as possible. The time taken to complete this test was also recorded in s. According to the 5XSST, two groups were formed as the following: 5XSST < 12.9 s and 5XSST ≥ 12.9 s.

The Flamingo test was used to assess balance, where participants were asked to stand on one leg, lifting the other so that the foot was resting against the knee of the standing leg for 30 s, without using support or losing balance. If the participants touched the ground with their lifted foot or hands, the test was evaluated as positive.

### 2.4. Statistical Analysis

Statistical analysis was conducted using SPSS version 23 with a significance level set as *p* < 0.05. Descriptive statistics, including the mean, SD, minimum, maximum, range and confidence interval (CI) were computed for all variables. The independent sample *t*-test was used to analyze statistical differences for continuous variables between two groups and one-way ANOVA for more than two. The results are presented in tables, bar charts, box plot diagrams and scatter plots.

## 3. Results

**Figure 1:**
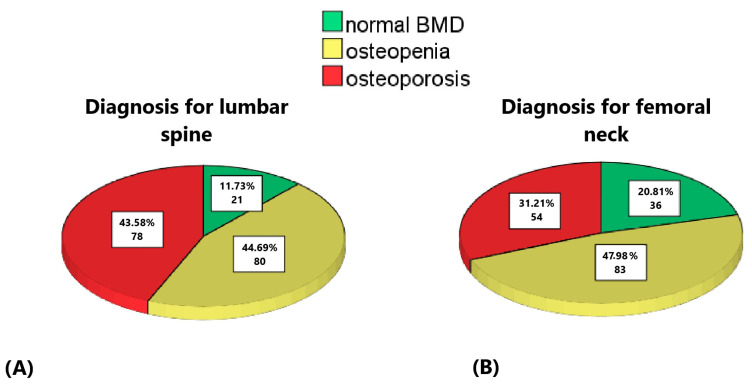
Distributions of the subjects with normal BMD (green), osteopenia (yellow) and osteoporosis (red): (**A**) for the lumbar spine scans, (**B**) for the femoral neck scans.

We assessed 182 women with a mean age of 70 years (yrs., range 60–88 yrs.). Their mean weight was 71 kg (40–120 kg), height 154.5 cm (100–182 cm) and BMI 29.6 kg/m^2^ (17.3–47.5 kg/m^2^). A total of 179 of the participants underwent a REMS scan of the lumbar spine. The REMS-based BMD averaged 0.810 kg/cm^2^ (0.585–1.258 kg/cm^2^), with a mean T-score of −2.2 SD, classifying 78 women (43.6%) as osteoporotic and 80 (44.7%) as osteopenic (Figure 1A). The femoral neck REMS-based BMD averaged 0.643 kg/cm^2^ (0.347–1.060 kg/cm^2^), with a T-score of −1.9 SD, resulting in 54 (31.2%) osteoporotic and 83 (48%) osteopenic diagnoses (Figure 1B). The mean FRAX value for MOF was 17.01% ± 0.69% (range 0.67–56.76%) and that for HF was 5.05% ± 5.11% (range 0.60–35.53%). Both mean FRAX values showed an intermediate risk of fractures in 10 years. HGS averaged 18.4 kg for both hands. The mean TUG time was 10 s and the 5XSST time 11 s (Table 1).

**Figure 2:**
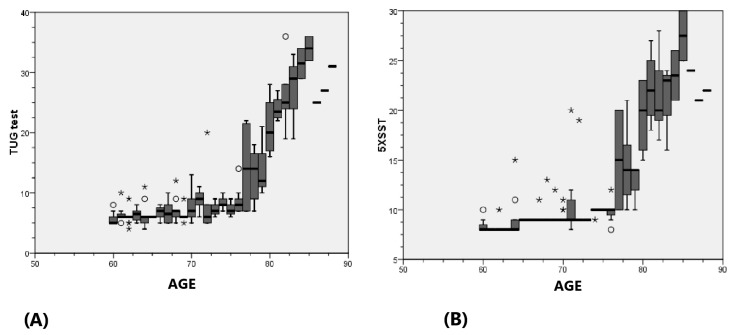
(**A**) Mean TUG in different age decades; (**B**) Mean 5XSST results in different age decades. ∘ outlier, * extreme outlier.

**Figure 3:**
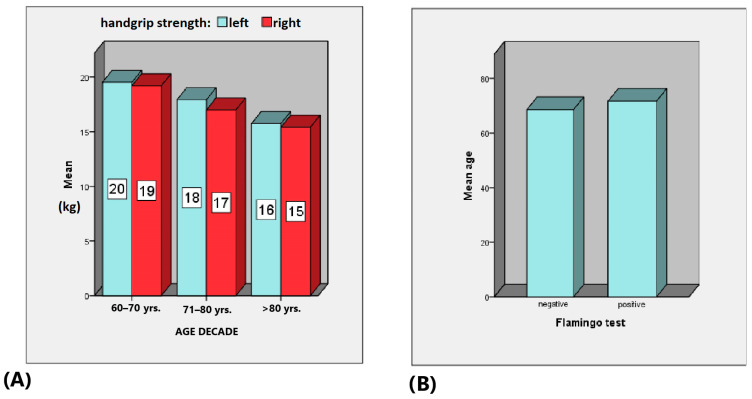
(**A**) Mean muscle strength of the left hand (blue) and muscle strength of the right hand (red) between the different age decades; (**B**) Mean age of subjects according to the Flamingo test.

TUG times increased significantly with age from 6.39 s (60–70 years) to 26.21 s (over 80), *p* < 0.001 with CI [8.95, 11.08] (Figure 2A). The 5XSST result times also increased with age, showing similar trends, *p* < 0.001 with CI [10.48, 11.93] (Figure 2B). HGS differed significantly (*p* < 0.001) across age decades, and subjects with a negative Flamingo test result were significantly (*p* = 0.004) younger (69 years) than those with positive test results (Figure 3).

**Figure 4:**
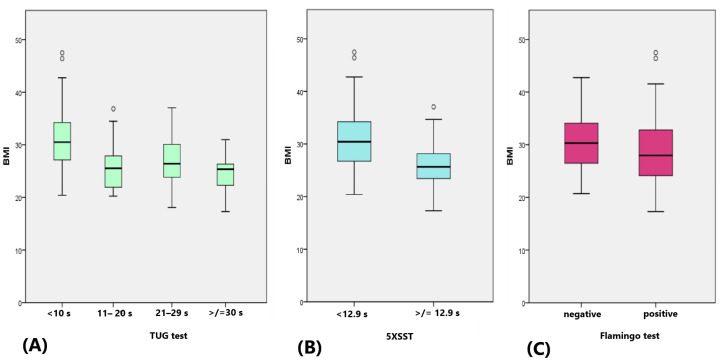
Comparison of BMI between the groups according to the (**A**) TUG, (**B**) 5XSST and (**C**) Flamingo test. ∘ outlier.

BMI varied significantly among the TUG groups, with lower TUG times associated with higher BMI, *p*-value < 0.001, CI [30.0, 31.8] (Figure 4A). The mean BMI in the group with 5XSST < 12.9 s (30.6 kg/m^2^) was significantly higher than that of the group with 5XSST ≥ 12.9 s (26.1 kg/m^2^), *p* < 0.001, CI [28.8, 30.4] (Figure 4B). Mean BMI did not differ significantly between the groups according to the Flamingo test, *p* = 0.121 (Figure 4C).

**Figure 5:**
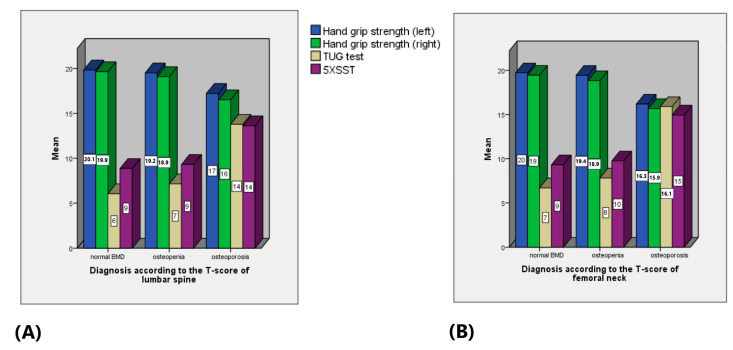
Mean HGS of the left (blue) and right hand (green), TUG test (yellow) and 5XSST (purple) between the groups with normal BMD, osteopenia and osteoporosis according to the BMD of the (**A**) lumbar spine and (**B**) femoral neck.

The mean HGS differed significantly between the groups with a normal BMD, osteopenia and osteoporosis according to the REMS-based BMD of the lumbar spine (*p* = 0.021 with CI [9.08, 9.45 kg] for the left hand and *p* = 0.03 with CI [8.79, 9.22 kg] for the right hand). The mean TUG and 5XSST times of the subjects with osteoporosis were significantly higher than those with osteopenia and normal BMD, *p* = 0.024 with CI [8.86, 10.94 s] for the TUG test and *p* = 0.012 with CI [10.42, 11.83 s] for the 5XSST (Figure 5A). Similar findings were observed according to the diagnosis of the femoral neck (Figure 5B).

**Figure 6:**
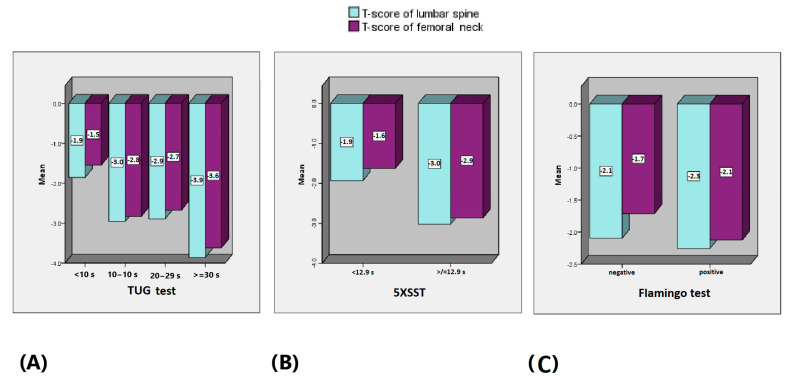
Mean T-scores of the lumbar spine (light blue) and femoral neck (purple) between the groups according to the (**A**) TUG test, (**B**) 5XSST and (**C**) Flamingo test.

The mean T-score of the lumbar spine of the subjects was significantly lower in the TUG group ≥ 30 s (−3.9 SD) than the groups 20–29 s (−2.9 SD), 10–19 s (−2.9 SD) and < 10 s (−1.9 SD), *p* = 0.001 with CI [−2.3, −1.9]. The mean T-score of the femoral neck of the subjects was significantly lower in the TUG group ≥ 30 s (−3.7 SD) than the groups 20–29 s (−2.7 SD), 10–19 s (−2.8 SD) and < 10 s (−1.5 SD), *p* = 0.002 with CI [−2.1, −1.7] (Figure 6A). The mean T-score of the lumbar spine of the subjects was significantly lower in the group with 5XSST ≥ 12.9 s (−3.0 SD) than 5XSST < 12.9 s (−1.9 SD), *p* = 0.001, CI [−2.1, −1.7]. The mean T-score of the femoral neck of the subjects was significantly lower in the group with 5XSST ≥ 12.9 s (−2.9 SD) than 5XSST < 12.9 s (−1.6 SD), *p* = 0.002, CI [−2.3, −1.9] (Figure 6B). The mean T-score of the lumbar spine of the subjects did not differ significantly between the groups with a positive Flamingo test result (−2.3 SD) and negative Flamingo test (−2.1 SD), *p* = 0.417, CI [−2.3, −1.9]. The mean T-score of the femoral neck of the subjects was significantly lower in the group with a positive Flamingo test result (−2.1 SD) than those with a negative Flamingo test result (−1.7 SD), *p* = 0.007, CI [−2.1, −1.7] (Figure 6C).

According to the 5XSST, the count of the women in the group with 5XSST < 12.9 s was 143 and the count in the group 5XSST ≥ 12.9 s was 39. The groups according to the TUG test were distributed as the following: with TUG < 10 s—134 subjects, with TUG 11–20 s—35 subjects, with TUG 21–29—8 subjects and with TUG ≥ 30 s—5 subjects. According to the Flamingo test, 98 women had a negative result and 84 women had a positive result.

**Figure 7:**
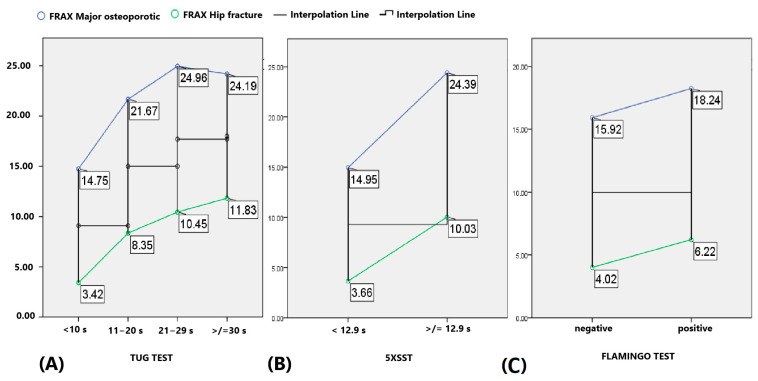
Differences in FRAX MOFs (blue line) and FRAX HFs (green line) between the groups according to the (**A**) TUG, (**B**) 5XSST and (**C**) Flamingo test.

Both FRAX results for the MOF and for HF differed significantly between the groups according to the TUG test, *p* < 0.001. The FRAX for the MOF was similar in the group with TUG ≥ 30 s (24.19%) and in the group with TUG 21–29 s (24.96%), but it was lower in the groups with TUG 11–20 s (21.67%) and TUG < 10 s (14.75%). The FRAX for the HF increased from the TUG group < 10 s (3.42%) to the group with TUG ≥ 30 s (11.83%) (Figure 7A). The FRAX for major MOFs differed significantly between the groups according to the 5XSST, *p* < 0.001, CI [15.65, 18.36%]. The FRAX for HFs differed significantly between the groups according to the 5XSST, *p* < 0.001, CI [4.29–5.80]. Subjects with a 5XSST result < 12.9 s had a lower FRAX for the MOF (14.95%) and FRAX for the HF (3.66%) than those with a 5XSST result ≥ 12.9 s (24.39% for the FRAX MOF and 10.03% for the FRAX HF) (Figure 7B). The FRAX for the MOF did not differ significantly between the groups according to the Flamingo test, *p* = 0.092, CI [15.6, 18.36%]. In contrast, the FRAX for the HF differed significantly between the groups according to the Flamingo test, *p* = 0.004, CI [4.29, 5.80%]. Subjects with a negative Flamingo test result had a lower FRAX for the HF (4.02%) than those with a positive Flamingo test result (6.22%) (Figure 7C).

## 4. Discussion

In our study, 78 women (43.6%) had osteoporosis of the spine, and 54 women (31.8%) of the femoral neck, consistent with the literature, indicating increased osteoporosis risk in postmenopausal women due to estrogen and vitamin D deficiency. Additionally, 80 women (44.7%) were classified as osteopenic at the lumbar spine and 83 (48%) at the femoral neck, suggesting that approximately 88% of participants had low bone mass, raising public health concerns. In such cases, nutritional and lifestyle interventions may help prevent further BMD loss.

We observed a significant increase in mean TUG and 5XSST times with age, indicating declining functional mobility, which aligns with previous studies [28,29]. There were also significant differences in the HGS and balance across age groups, reinforcing the link between age, muscle strength and balance, which are crucial for independence. These findings also corroborate previous research [30,31,32].

Mean BMI varied based on TUG performance, suggesting that a higher BMI may positively influence mobility, although BMI did not significantly differ in the Flamingo test. A similar trend was observed with the 5XSST. Previous published studies are controversial regarding the relation of BMI to balance and falls as BMI is not the best indicator of obesity [33,34,35,36,37]. In contrast to our findings, several studies found that overweight and obesity negatively affect an individual’s mobility [38,39,40]. This could have been caused by the lack of information in our study about the BMI history of the subjects, BMI categorization (underweight, normal, overweight and obese) and total body DXA scan results which could have better described their muscle mass, fat mass and bone mass. On the other hand, Yoo et al., in a longitudinal study, identified that high BMI had a protective effect on the reduction in muscle mass in older men and women. However, obesity parameters including BMI, waist circumference and percentage of body fat were positively correlated with a lower incidence of sarcopenia only in the female population [41]. These results support our findings. Furthermore, Kıskaç et al., defined that the optimal BMI range is 31–32 kg/m^2^ for geriatric females to avoid decreased functional capacity and balance, as well as reduction in muscle strength [42]. Future studies are needed to further investigate the effect of BMI on physical performance in the geriatric population.

HGS decreased with lower REMS-based BMD, aligning with the findings from DXA and ultrasound studies of the calcaneus [43,44,45]. Furthermore, TUG and 5XSST times worsened with decreased REMS-based BMD of the spine and hip. The lack of the expected trend in the groups with TUG times of 20–29 s and 10–19 s for the lumbar spine may be due to statistical variability, for example, there could have been some individuals in the groups who had much worse bone density but still performed relatively better in the TUG test, leading to a flattened or unexpected pattern in the data. A comparable trend was observed in the results for the femoral neck. These results indicated that the T-score did not deteriorate with increasing TUG time, but instead worsened after a certain threshold. Similar findings were reported by previous studies, which identified osteoporosis through a DXA scan [46,47]. There are no studies that have reported a decreased HGS and functional decline with worsening of the REMS-based BMD of the spine and hip assessed with REMS. Further studies could explore the underlying mechanisms linking muscle strength and BMD.

Notably, faster 5XSST performance correlated with a lower FRAX for the major osteoporotic fractures (MOF) and for the hip fractures (HF). Similarly, TUG times under 10 s indicated a significantly lower fracture risk (FRAX for MOF: 14.75% vs. 24.19% for ≥ 30 s). This suggests that higher body strength and mobility are protective against fractures. The TUG and 5XSST results were also associated with increased fracture risk in previous studies [48,49]. A cross-sectional study found that physical decline and frailty associated with aging, such as decreased hand grip strength, are highly correlated with a decreased number of teeth and occlusal force [50]. These results show that future studies are needed to investigate this aspect with regard to REMS-based BMD and fracture risk.

### Strengths and Weaknesses of This Study

Strengths: This study’s prospective observational design enhanced causal inference. The cohort of 182 geriatric women allowed for a robust statistical analysis. Established tools like the FRAX, TUG and 5XSST ensured reliable measurements, supporting the credibility of our findings. This study also uniquely explored relationships between physical performance and BMD using REMS.

Weaknesses: Limitations included potential biases in self-reported demographic data and limited participant diversity. The absence of data on medication use and other pathologies limited the ability to account for its potential impact on fracture risk, BMD and other outcomes, which may have influenced the generalizability of the findings to patients with different treatment regimens. The selected tests may not have encompassed all aspects of mobility and functional ability, which could have skewed the overall health assessments.

## 5. Conclusions

This study revealed strong associations between physical performance and fracture risk, highlighting the need to improve mobility and strength in older adults. By shedding light on this issue, we hope to contribute to a more comprehensive understanding of the factors that influence skeletal health in older women, ultimately aiding in the development of targeted interventions to reduce fracture risk and improve overall quality of life. Rehabilitation programs focused on strength and mobility may be essential for fracture prevention.

## Figures and Tables

**Table 1:** The subjects’ characteristics.

	Mean	Median	Minimum	Maximum	Standard Deviation	Standard Error of the Mean
Age	70	69	60	88	7	1
Weight	71.0	69.0	40.0	120.0	14.6	1.1
Height	154.5	155.0	100.0	182.0	8.3	0.6
BMI	29.6	29.3	17.3	47.5	5.6	0.4
REMS-based BMD L1	0.712	0.697	0.427	1.134	0.127	0.01
REMS-based BMD L2	0.788	0.778	0.567	1.251	0.123	0.01
REMS-based BMD L3	0.844	0.829	0.578	1.284	0.121	0.01
REMS-based BMD L4	0.868	0.859	0.632	1.315	0.117	0.01
Total REMS-based BMD	0.810	0.798	0.585	1.258	0.117	0.01
Total REMS-based T-score	−2.2	−2.3	−4.2	3.0	1.1	0.1
Total REMS-based Z-score	−0.1	−0.3	−1.5	2.8	0.8	0.1
FRAX for major osteoporotic	17.01	15.13	0.67	56.76	9.17	0.69
FRAX for hip fracture	5.05	3.16	0.60	35.53	5.11	0.38
REMS-based BMD of femoral neck	0.643	0.637	0.347	1.060	0.126	0.01
REMS-based T-score of femoral neck	−1.9	−1.9	−4.5	1.2	1.1	0.1
REMS-based Z-score of femoral neck	−0.1	−0.2	−2.1	3.0	0.9	0.1
REMS-based BMD of trochanter	0.803	0.800	0.456	1.214	0.135	0.01
REMS-based T-score of trochanter	−0.1	−1.0	−3.6	2.1	0.9	0.07
REMS-based Z-score of trochanter	0.2	0.2	−1.8	3.4	0.9	0.1
REMS-based total BMD of hip	0.787	0.783	0.442	1.266	0.141	0.01
REMS-based total T-score of hip	−1.3	−1.3	−4.1	1.9	1.1	0.1
REMS-based total Z-score of hip	0.2	0.1	−2.0	3.4	0.9	0.1
Hand grip strength (left)	18.4	20	8	20	3	0
Hand grip strength (right)	18.4	20	6	20	3	0
TUG test	10	7	4	36	7	1
5XSST	11	9	8	30	5	0

BMI—body mass index; REMS—radiofrequency echographic multispectrometry, BMD—bone mineral density; FRAX—Fracture Risk Assessment Tool; TUG—Timed Up and Go; 5XSST—Five Times Sit-to-Stand Test.

## Data Availability

The datasets presented in this article are not publicly available due to the inclusion of information that could compromise the privacy of research participants.
